# The epidemiology of typhoid fever in the Dhulikhel area, Nepal: A prospective cohort study

**DOI:** 10.1371/journal.pone.0204479

**Published:** 2018-09-27

**Authors:** Neta Petersiel, Sudeep Shresta, Rajendra Tamrakar, Rajendra Koju, Surendra Madhup, Ashish Shresta, TRS Bedi, Niv Zmora, Yael Paran, Eli Schwartz, Ami Neuberger

**Affiliations:** 1 Unit of Infectious Diseases–Tropical Diseases & Travel Medicine, Internal Medicine B, Rambam Medical Center, Haifa, Israel; 2 Dhulikhel Hospital, Kathmandu University Hospital, Dhulikhel, Nepal; 3 Tel Aviv Sourasky Medical Center, Tel Aviv, Israel; 4 Sackler Faculty of Medicine, Tel Aviv University, Tel Aviv, Israel; 5 The Center for Geographic Medicine and Tropical Diseases, the Chaim Sheba Medical Center, Tel Hashomer, Israel; 6 Bruce Rappaport Faculty of Medicine, Technion, Haifa, Israel; Laboratoire National de Santé, LUXEMBOURG

## Abstract

**Introduction:**

Typhoid fever (TF) continues to cause considerable morbidity and mortality in Nepal, but only limited epidemiologic data is available about TF outside Kathmandu.

**Methods:**

As part of an interventional trial, we performed a prospective cohort study of bacteremic TF patients in Dhulikhel Hospital between October 2012 and October 2014. Demographic, epidemiological, clinical, and microbiologic data were recorded.

**Results:**

116 bacteremic typhoid patients were included in the study. Most were young, healthy, adults (mean age 27.9±12 years), 41.4% of whom were female. More than 70% of patients were employed in non-manual services or were university students. *Salmonella* Typhi accounted for 64/115 (55.7%) of all isolates, while *Salmonella* Paratyphi accounted for 51/115 (44.3%), of which 42 were Paratyphi A and 9 Paratyphi B. A significant proportion of TF cases occurred also during the dry season (48/116, 41.6%). The clinical presentation of *Salmonella* Typhi and Paratyphi infections was similar, except for a greater proportion of arthralgia in patients with *Salmonella* Typhi. Most *Salmonella* Typhi and Paratyphi isolates were resistant to nalidixic acid and susceptible to older antibiotics. One *Salmonella* Paratyphi isolate was resistant to ceftriaxone.

**Conclusions:**

TF remains common in the Dhulikhel area, even among those with a high level of education. Public health measures aimed at reducing the incidence of TF in the Dhulikhel area are warranted. The relative burden of TF caused by *Salmonella* Paratyphi is rising; a vaccine with activity against *Salmonella* Paratyphi is needed. Since *Salmonella* Paratyphi B was more prevalent in this cohort than in large cohorts of patients from Kathmandu, it is likely that there are significant regional variations in the epidemiology of TF outside Kathmandu.

## Introduction

Typhoid (enteric) fever (TF) is caused by the pathogens *Salmonella enterica* subsp. *enterica* serovars Typhi or Paratyphi (*Salmonella* Typhi/Paratyphi), and is transmitted via a fecal-oral route. The burden of TF is highest in the Indian subcontinent, where it peaks during the rainy season, from June to August[1] [2].

In Nepal, TF is common in all populated areas: in the low-lying southern Terrai region, as well as in the Kathmandu valley. In recent years, increased urbanization, a growing population density within the major cities, and the widespread lack of access to clean water and food have led to a very high prevalence of TF throughout the country [3]. In fact, *Salmonella* is by far the most common single pathogen isolated in blood cultures in Nepal [4][5][6][7][8]. Prior reports from Kathmandu link low socioeconomic status and unsanitary living conditions to higher prevalence of TF[9]. The annual incidence of TF in this area was calculated at 3.78 per 1000 households.[1] In the hilly town of Dhulikhel, situated 30 kilometers southeast of Kathmandu, previous reports have shown high prevalence of TF, and high rates of nalidixic acid and fluoroquinolone resistance[5][10][11][12]. There is limited data describing current epidemiology of TF from this area, and from other regions outside Kathmandu.

There have been tremendous socioeconomic changes in Nepal in recent years, with impact on the epidemiology of TF [3]. Our aim is to describe the epidemiological characteristics of bacteremic patients with TF in Dhulikhel, Nepal.

## Methods

Findings were based on a prospective cohort of patients with blood culture positive TF, derived from a comparative trial of mono versus dual antimicrobial therapy for TF, who were treated in Dhulikhel Hospital or its outpatient clinic between October 2012 and October 2014 (NCT02224040). The hospital serves a population of about 20,000 people in Dhulikhel itself, and functions as a community medical center for the neighboring towns and surrounding countryside. Since treatment is subsidized and medical standards are relatively high, patients from faraway villages or from towns that have their own medical centers often present to Dhulikhel Hospital's emergency room and outpatient clinic. Inclusion criteria for the study were fever or a history of fever, and a positive blood culture for *Salmonella* Typhi or Paratyphi. We excluded patients with no positive blood culture.

Trained staff administered a structured questionnaire, which included demographic, epidemiological, and clinical data. A physician performed a physical exam, which included assessment of vital signs, and cardiovascular and abdominal exams. Blood was cultured in the hospital's microbiology lab using BACTEC™ instrumented blood culture systems. Bacteria were identified using standard biochemical tests, and serotyping was performed by slide agglutination tests using specific Salmonella anti-Vi and anti-D antisera. Antibiotic susceptibilities were determined using the Kirby-Bauer disc diffusion method. We used breakpoints defined by the Clinical Laboratory Standards Institute (CLSI) at the time. Since there were no clear breakpoints to define resistance to azithromycin before 2015, we did not include susceptibility reporting for this antibiotic.

Dichotomous variables were analyzed with chi-square or Fisher’s exact test, as appropriate. Continuous variables were compared with the use of Student’s t-test for parameters with normal distribution, and the Mann-Whitney test for variables with abnormal distribution. Multiple groups were compared by one-way ANOVA. Data are presented as means ± SD; p < 0.05 was considered statistically significant.

The study protocol was reviewed and approved by the Kathmandu University School of Medical Sciences’ Institutional Review Committee (KUSMS/IRC). Written, informed consent was obtained from all participants or from the parents of children younger than 18 years, after they were given a detailed explanation in Nepalese.

## Results

During the study period, 116 patients with bacteremic TF were screened for the comparative trial; all were included in this epidemiologic study. There were 67 (57.8%) males, and 48 (41.4%) females (gender of one participant was not recorded). The average age of adult patients was 27.9 ± 11.5. The average age of pediatric patients was 12.0 ± 5.1. Cases were unevenly distributed throughout the hospital catchment area. Forty-three out of 98 (43.9%) patients with recorded addresses lived in villages, the others in towns or cities. TF was diagnosed in patients residing in the towns of Bhaktapur (14/98, 14.2%), Panauti (14/98, 14.2%), Banepa (9/98, 9.1%), and Dhulikhel itself (10/98, 10.2%); other cases occurred in neighboring villages, with notably more cases occurring north of Dhulikhel. Patients’ occupations were recorded for 87 adult patients. There were 40 (46%) students, 13 (14.9%) homemakers, 12 (13.8%) farmers, and 22 (25.3%) patients employed in various non-manual services (Table 1).

**Table 1:** Demographic and microbiologic characteristics of patients with TF in Dhulikhel.

	Patients with typhoid fever in Dhulikhel (N = 116)
**demographic**	
female (%)	48 (41.4)
Male (%)	67 (57.7)
missing	1 (0.8%)
Age, adults (years ± standard deviation)	27.9 ± 11.5 (range 18–81)(N = 101)
Age, children (years ± standard deviation)	12.0 ± 5.1 (range 2.5–17)(N = 13)
Occupation	farmers 12 (13.8%)
	students 40 (46%)
	non-manual services 22 (25.3%)
	unknown 14 (13.8%)
Occurring during the rainy season (June–September, %)	58.4%
**microbiology**	
*Salmonella* Typhi (%)	64/116 (55.7%)
*Salmonella* Paratyphi A (%)	42/116 (36.5%)
*Salmonella* Paratyphi B (%)	9/116 (7.8%)

Only eight participants had an underlying illness, which reflects the young age of patients with TF. Fifty-four patients (46.6%) were hospitalized, while 62 (53.4%) were treated as outpatients. No patients had gastrointestinal perforation, gastrointestinal hemorrhage, and no mortalities were reported.

*Salmonella* Typhi accounted for 64/116 (55.7%) of all isolates, while *Salmonella* Paratyphi accounted for 52/116 (44.3%); 42 of the latter were identified as *Salmonella* Paratyphi A, and 9 as *Salmonella* Paratyphi B (one isolate was only identified as *Salmonella* Paratyphi without serovar).

Relatively few isolates were resistant to “old” antibiotics, such as ampicillin (18/114, 15.7%), trimethoprim-sulphamethoxazole (2/115, 1.7%), and chloramphenicol (2/115, 1.7%). Conversely, resistance to nalidixic acid (105/112, 93.7%), and to ciprofloxacin (102/115, 87.9%) were widespread (Table 2). Of great concern one isolate of *Salmonella* Paratyphi A was resistant to ceftriaxone.

**Table 2:** Antibiotic susceptibility pattern of *Salmonella enterica* serovars Typhi / Paratyphi in bacteremic patients.

Antibiotic type	*Salmonella enterica* serovar Typhi (% susceptible)	*Salmonella enterica* serovar Paratyphi A (% susceptible)	*Salmonella enterica* serovar Paratyphi B (% susceptible)
**Ciprofloxacin**	9/63 (14)	4/42 (9.5)	0/9 (0)
**Nalidixic acid**	5/62 (7.8)	2/41 (4.7)	0/9 (0)
**Ampicillin**	60/62 (93.7)	30/42 (71.4)	6/9 (66.6)
**Trimethoprim/ Sulfamethoxazole**	61/63 (95.3)	42/42 (100)	9/9 (100)
**Chloramphenicol**	62/63 (96.8)	41/42 (97.6)	9/9 (100)
**Ceftriaxone**	64/63(100)	41/42 (97.6)	9/9 (100)

TF caused by *Salmonella* Typhi and Paratyphi A occurred more commonly during the monsoon season between June and September [39/64 (60.9%), and 23/42 (54.8%), respectively]. Still, a significant proportion (41.6%) of cases caused by either *Salmonella* Typhi or *Salmonella* Paratyphi occurred during the dry season. There was no seasonal pattern in cases caused by *Salmonella* Paratyphi B; only three out of nine were contracted during the rainy season, although the number of cases was small.

In 108/116 clinical characteristics and lab results were recorded. We observed no differences in the clinical characteristics of *Salmonella* Typhi and Paratyphi infections (Table 3), the only exception being arthralgia, which was more common in patients infected by *Salmonella* Typhi as compared with patients infected by *Salmonella* Paratyphi (11/57 versus 2/51, respectively, p = 0.01) (Table 3). There were no additional differences in the demographic data, medical history, and blood-test results when patients with *Salmonella* Typhi and *Salmonella* Paratyphi were compared (p-values for all other comparisons > 0.05).

**Table 3:** Clinical characteristics of *Salmonella* Typhi and Paratyphi infections.

characteristics	*Salmonella* Typhi N = 57 (%)	*Salmonella* Paratyphi N = 51 (%)	P value
**Clinical presentation**			
Anorexia	29 (50.8)	20 (39.2)	0.22
Vomiting	6 (10.5)	6 (11.7)	0.83
Abdominal pain	6 (10.5)	10 (19.6)	0.18
Diarrhea	16 (28)	7 (13.7)	0.07
Constipation	4 (7)	8 (15.6)	0.19
rash	1 (1.7)	1 (1.9)	0.93
Headache	45 (78.9)	42 (82.3)	0.65
Cough	9 (15.7)	6 (11.7)	0.54
Fever	57 (100)	51 (100)	1
Myalgia	12 (22)	10 (19.6)	0.85
Arthralgia	11 (19.2)	2 (3.9)	0.01
**Laboratory values**			
Leukocytes (*X*10^3^/*μL*) median (min-max)	7350 (1180–14800)	7400 (2900–18000)	0.99
CRP (mg/L) Median (min-max)	38 (5–120)	28 (5–120)	0.15

## Discussion

This report summarizes epidemiologic and clinical data of patients with bacteremic TF outside of Kathmandu. The geographic distribution of 116 patients in the Dhulikhel area was uneven, and the disease occurred not only among the poor. *Salmonella* Paratyphi accounted for nearly half the cases, while *Salmonella* Paratyphi B constituted a sizable portion (17.6%) of Paratyphi cases. To the best of our knowledge, this is the first such report. Seasonality during the rainy season was pronounced in cases caused by *Salmonella* Typhi and Paratyphi A, but not in cases caused by *Salmonella* Paratyphi B, although numbers were small.

TF is considered a disease more prevalent among the poor, with more than 50% of those affected having a low income [9]. It is surprising that the majority of our patients did not belong the lower socioeconomic part of the Nepalese population, but rather held non-manual jobs or were students. Most previous studies were conducted in Kathmandu [9,11,13,14], and it is likely that the epidemiology of TF is different outside urban centers. Another possible explanation to this observation is a referral bias, as poorer patients may have found it too expensive to travel to Dhulikhel.

The uneven geographic distribution is noteworthy. Although most patients in the hospital reside in Dhulikhel itself, the vast majority of patients with TF were from other towns or from smaller villages. It seems that a relatively high percentage of patients came from the towns of Panauti and Banepa. This observation suggests typhoid “hotspots” in the region, and has important public-health implications.

In previous studies from Nepal, *Salmonella* Typhi was two to four times more common than *Salmonella* Paratyphi A[7] [15] [1], although the relative burden of the latter has increased gradually over the past two decades[8][1]. Our data shows that *Salmonella* Paratyphi infection is common in the Dhulikhel area, accounting for nearly half of all cases. In previous reports, *Salmonella* Paratyphi B was a rare cause of bacteremia. Only about 3% of the Nepalese population showed evidence of past infections with this serovar[16]. In some reports no cases were detected at all, and in the largest cohort published to date, this bacterium accounted for about 0.1% of 19,857 cases of *Salmonella* bacteremia in Patan Hospital in Kathmandu. [7][8][1]. The relative proportion of *Salmonella* Paratyphi B in our cohort is roughly 70-fold higher than those reported from Kathmandu (9/116, 7.8%), and accounts for 17.6% of all Paratyphi isolates. These cases of *Salmonella* Paratyphi B were not connected geographically or temporally. We assume that there may be regional and seasonal variations in the occurrence of the various *Salmonella enterica* serovars within Nepal.

In line with recent reports from Nepal [6–8,11,15,17], we observed very high levels of nalidixic acid and fluoroquinolone resistance, and relatively low rates of resistance to older drugs such as chloramphenicol, ampicillin, and trimethoprim sulfamethoxazole. Ciprofloxacin is indeed often sold in pharmacies without medical prescriptions throughout the country [18]. There was one patient with ceftriaxone-resistant *Salmonella*; several such cases have been reported in recent years, although the vast majority of isolates in most studies were susceptible [7][19]. The appearance of ceftriaxone resistance, possibly mediated by acquisition of β-lactamases, is worrisome, as ceftriaxone is the first-line choice for hospitalized patients with severe TF [20]. As resistance to ampicillin, trimethoprim-sulphamethoxazole, and chloramphenicol was generally low, the use of older medications may be required to curb resistance to newer antibiotics. Another approach might be the administration of dual antibiotic therapy which was reported to shorten fever clearance time, and shorten the duration of bacteremia [21][22]

Although the official WHO webpage still claims "paratyphoid" fever to be a milder disease, clinical features of TF, caused by either *Salmonella* Typhi or Paratyphi, have been reported to be similar by some studies in locals and travelers[22] [13] [23]. Patients infected by either serovar did indeed have a similar clinical presentation, with the exception of arthralgia, which was much more common in infections caused by *Salmonella* Typhi. Larger cohorts are needed to verify this observation.

The Strategic Advisory Group of Experts on immunization (SAGE), which advises the WHO, recently recommended conjugate TF vaccine for routine use in pediatric populations over six months of age living in endemic areas [24].

The growing share of TF caused by *Salmonella* Paratyphi over the last two decades might limit the public health impact of a conjugated vaccine, and may lead to a further relative increase in "paratyphoid" fever cases[25]. In this sense, it is important to remember that the introduction of the conjugate vaccine does not absolve the country and its international partners from ensuring that the Nepalese population has access to safe food and water.

There is some evidence that a combination of parentreral Vi-capsular polysaccharide vaccine with an oral whole-cell *Salmonella* Ty21a vaccine enhances specific response, and cross-reacts with both *Salmonella* Paratyphi and non-typhoidal *Salmonella*[26]. A trial of combined oral and parentral vaccines is warranted [27]. Such trial could be conducted in one of the suspected TF hotspots mentioned above.

The study has a number of limitations. The small sample size and a referral bias of some patients (e.g. those who could afford the costs of travel to the hospital or were physically fit enough to walk) are obvious. Recruitment rates were low among pediatric patients, and this fact makes our conclusions more relevant to adult patients. Since blood cultures have limited sensitivity, many cases of TF were invariably overlooked. We have assumed infection was acquired in the place of residence, where typhoid fever is usually acquired. However, we did not carry out individual case investigations, so the geographical distribution of TF cases may not be completely accurate. One clear advantage of this study is the inclusion of patients with proven *Salmonella* bacteremia only, as many clinical diagnoses of TF were proven to be non-TF[14].

This is one of the few epidemiologic descriptions of TF in Nepal, outside of Kathmandu, in the past decade. Compared to previous studies, we found relatively more cases caused by *Salmonella* Paratyphi, an unexpected number of cases caused by *Salmonella* Paratyphi B, a unique pattern of antibiotic resistance, and suspected TF hotspots in the Dhulikhel area. In light of these regional variations in the epidemiology of TF, the rapid socioeconomic changes Nepal is going through, and the expected introduction of conjugated vaccines, it is time an active surveillance system for typhoid fever cases in Nepal be established.

## Supporting information

S1 File (DOCX)
