# Supplementary material for: The epidemiology of typhoid fever in the Dhulikhel area, Nepal: A prospective cohort study
Source: PLoS One. 2018 Sep 27;13(9):e0204479. doi: 10.1371/journal.pone.0204479 (PMC6160059; doi:10.1371/journal.pone.0204479)
Supplement: S1 File — (DOCX) [file pone.0204479.s001.docx]

**Working Proforma**

Date: ...................

Hospital number: .......................... Study number: .........................

Date of admission: ........................ Date of discharge: ....................

Duration of hospital stay: ........days

Patient admitted from: Emergency 🞏 OPD 🞏

1. Identification:

Name: .........................................................

Age: ................ years Sex: Male 🞏 Female 🞏

Occupation: ................................. Marital status: .................................

Address: ....................................................................... Mobile number: ………………………

1. Presenting complaints:
   1. Fever: Duration: ...... days
   2. Chills 🞏
   3. Headache 🞏
   4. Cough 🞏
   5. sweating 🞏
   6. Myalgias 🞏
   7. Malaise 🞏
   8. Arthralgia 🞏
   9. Anorexia 🞏
   10. Nausea 🞏
   11. Vomiting 🞏
   12. Abdominal pain 🞏
   13. Diarrhoea 🞏
   14. Constipation 🞏
   15. Others 🞏
2. Use of antibiotics prior to admission: .........days Name of antibiotic …………………..
3. Past history of typhoid fever: Yes 🞏 No 🞏
4. Any significant underlying illness: ............................................
5. Menstrual and obstretic history: LMP: ...........
   Pregnant: Yes 🞏 No 🞏 Lactating: Yes 🞏 No 🞏
6. Allergy to any drugs:Yes 🞏 No 🞏 if yes specify allergy: .......................
7. Paracetamol intake: ........days
8. Vitals:

| Date |  | |  | |  | |  | |  | |  | |  | |  | |  | | |
| --- | --- | --- | --- | --- | --- | --- | --- | --- | --- | --- | --- | --- | --- | --- | --- | --- | --- | --- | --- |
|  | am | pm | am | pm | am | pm | am | pm | am | pm | am | pm | am | pm | am | pm | am | pm |  |
| Heart Rate |  |  |  |  |  |  |  |  |  |  |  |  |  |  |  |  |  |  |  |
| Blood Pressure |  |  |  |  |  |  |  |  |  |  |  |  |  |  |  |  |  |  |  |
| Respiratoy Rate |  |  |  |  |  |  |  |  |  |  |  |  |  |  |  |  |  |  |  |
| Rectal Temperature |  |  |  |  |  |  |  |  |  |  |  |  |  |  |  |  |  |  |  |

1. Physical examination:

| Date |  |  |  |  |  |  |  |  |  |
| --- | --- | --- | --- | --- | --- | --- | --- | --- | --- |
| Rash |  |  |  |  |  |  |  |  |  |
| Hepatosplenomegaly |  |  |  |  |  |  |  |  |  |
| Abdominal tenderness |  |  |  |  |  |  |  |  |  |

1. Investigations:

| Date |  |  |  |  |  |  |  |  |  |
| --- | --- | --- | --- | --- | --- | --- | --- | --- | --- |
| Hb |  |  |  |  |  |  |  |  |  |
| TLC |  |  |  |  |  |  |  |  |  |
| DLC  (Neutrophils/Lymphocytes) |  |  |  |  |  |  |  |  |  |
| LFT |  |  |  |  |  |  |  |  |  |
| ESR/CRP |  |  |  |  |  |  |  |  |  |
| ECG |  |  |  |  |  |  |  |  |  |

|  | **Date** | **Sample** | **24 hrs** | **48 hrs** | **72 hrs** | **96 hrs** | **120 hrs** |
| --- | --- | --- | --- | --- | --- | --- | --- |
| On admission |  | Blood |  |  |  |  |  |
| On admission |  | Urine |  |  |  |  |  |
| On Day 3 |  | Blood |  |  |  |  |  |
| On follow up |  | Stool |  |  |  |  |  |

1. Culture findings:

1. Sensitivity: (S: sensitive, PS: Partially sensitive, R: Resistant)

| **Drug** | **Blood on admission** | **Blood on Day 3** | **Urine on admission** | **Stool On follow up** |
| --- | --- | --- | --- | --- |
| Ciprofloxacin |  |  |  |  |
| Ofloxacin |  |  |  |  |
| Ceftriaxone |  |  |  |  |
| Chloramphenicol |  |  |  |  |
| Amoxicillin |  |  |  |  |
| Cotromoxazole |  |  |  |  |
| Nalidixic acid |  |  |  |  |
| Azithromycin |  |  |  |  |
| Cefixime |  |  |  |  |

1. Treatment:

| Antibiotics | **Dose/Route** | **Starting date** | **Stopping date** | **Duration** | **Switchover to other antibiotics** |
| --- | --- | --- | --- | --- | --- |
| Azithromycin |  |  |  |  |  |
| Ceftriaxone |  |  |  |  |  |
| Azithromycin+Ceftriaxone |  |  |  |  |  |
| Cefixime |  |  |  |  |  |

1. Outcome:
   1. Cured (free from symptoms and signs) 🞏
   2. Improved 🞏
   3. Worsened 🞏
   4. Unchanged 🞏
   5. Died 🞏
2. Complications:
   1. Gastrointestinal bleeding 🞏
   2. Gastrointestinal perforation 🞏
   3. Relapse 🞏
   4. Adverse drug reactions 🞏 (specify reactions: …………………………………)
   5. Others 🞏
3. Condition on discharge:
   1. Improved 🞏
   2. Worsened 🞏
   3. Unchanged 🞏
4. Fever clearance time (<37.5C)............hrs
